# Xylazine Poisoning in Clinical and Forensic Practice: Analysis Method, Characteristics, Mechanism and Future Challenges

**DOI:** 10.3390/toxics11121012

**Published:** 2023-12-11

**Authors:** Tingting Mai, Youyou Zhang, Shuquan Zhao

**Affiliations:** 1 Faculty of Forensic Medicine, Zhongshan School of Medicine, Sun Yat-Sen University, Guangzhou 510275, China; maitt@mail2.sysu.edu.cn; 2 Guangdong Province Translational Forensic Medicine Engineering Technology Research Center, Guangzhou 510275, China; 3 Department of Geriatric Neurology, The Second Affiliated Hospital of Xi’an Jiaotong University, Xi’an 710004, China; tjf1289@126.com

**Keywords:** xylazine poisoning, clinical practice, forensic practice, characteristics, future challenge

## Abstract

Xylazine abuse is emerging globally, while the identification of xylazine lethal cases poses a great challenge in clinical and forensic practice. The non-specific symptoms delay the diagnosis and treatment of xylazine poisoning, the pathological changes and lethal concentration of xylazine in body fluid and organs of fatal xylazine poisoning cases are seldom reported and the other toxins detected in such cases complicate the role of xylazine in the cause of death. Therefore, we carefully reviewed related updated information on xylazine, summarized the knowledge from clinical and forensic perspectives and can thus provide a reference in such cases and throw light on further study in the field of xylazine poisoning.

## 1. Introduction

Xylazine is a strong agonist of α2 adrenergic receptors that could decrease the release of norepinephrine and dopamine from the brain and thus form a sedative effect [1]. In veterinary practice, it can achieve the required sedative effect used alone or combined with other narcotic drugs including ketamine [2]. The good sedative effect has made it popular in veterinary practice worldwide since the United States introduced it in the 1960s [3]. Generally, it is only legally available for animals with a veterinary prescription, so it is not listed as a controlled substance all over the world [4].

Xylazine can depress the central nervous system and respiratory functions, resulting in bradycardia, hypotension, and transient hyperglycemia; therefore, it has never been approved for human use [5,6]. Indeed, xylazine poisoning in humans was a rare thing at first, whereas, recently, xylazine has been emerging as a street drug [7]. At first, xylazine was used in drug-facilitated crimes such as sexual assault and robbery, but now it is more common in various street drugs across the US and is associated with drug overdose [5,6,7,8].

During the last 5 years, the incidence of xylazine involved in drug-related deaths in West Virginia increased by more than six-fold [9]. The increase in xylazine participation in rural states indicated the widespread and emerging public legal issues concerning the misuse and abuse of xylazine in the United States [9]. Recently, a xylazine-related death was first reported in Europe [10]. Indeed, xylazine poisoning is emerging in clinical and forensic practice; whereas most of them are case reports and they are often combined with other toxins or drugs, the pathological changes in xylazine poisoning cases have seldom been reported [10,11,12,13,14,15]. Therefore, the lethal concentration of xylazine in blood and other organs is far from identified and the underlying mechanism in the cause of death in xylazine-related deaths needs further study to be demonstrated.

To provide reference on xylazine poisoning, we carefully reviewed the latest updated information on it. Blood and urine were easy to acquire in clinical and forensic practice and the concentration of other drugs and toxins in these samples was well established in previous studies. Therefore, we not only summarized the symptoms of the victims in such cases, but also collected the blood and urine concentrations of xylazine in those cases. The articles involved in the present review were published before November 2023 on PubMed and CNKI. Xylazine was a constant keyword, and poisoning was added to focus on clinical and forensic interest. All the reported concentrations of xylazine were converted in ng/mL or ng/g to make a comparison.

## 2. Xylazine, Structures and Pharmacokinetics

### 2.1. Xylazine, Structures and Usage

Xylazine is a strong agonist of α2 adrenergic receptors, with a chemical name 2-(2,6-dimethylanilino)-5,6-dihydro-4h-1,3-thiazine. Its molecular formula is C12H16N2S with a 220.33 relative molecular mass. The chemical structure of xylazine is very similar to phenothiazines, tricyclic antidepressants and clonidine [16]. Since it was approved in 1960s, following comprehensive research including into its synthesis process, pharmacological properties, etc., its good sedative effect in veterinary practice has made it popular. It is provided in the form of hydrochloride in a solution containing 20 or 100 mg/mL, indicated by a free base. The routine administration of xylazine is intramuscular, intra-venous or subcutaneous; the intramuscular and subcutaneous injection of xylazine is absorbed quickly in equines. The dosages of xylazine vary from 0.55 to 8.8 mg/kg intramuscularly or 1.1 mg/kg a pound intravenously [17]. This dose could provide analgesia of 15 to 30 min and sedation of 1 to 2 h [18]. Generally, pharmacokinetics information about xylazine in humans is absent.

### 2.2. Pharmacokinetics

The intramuscular or subcutaneous injection of xylazine is absorbed quickly [19]. It produces a fast onset of action with a short duration. In addition, the intensity and duration of sedative or analgesic effects are proportional to the dose of drug used, and species also plays a role in the process of sedative effects [20]. An ordinary dose in a sheep can keep the animal quiet and asleep for 1~2 h, with analgesia for 15~30 min [18]. Cattle are the most sensitive animals to xylazine, and it is reported that the dose of xylazine used in cattle amounts to 1/10 of that in horses or dogs to achieve the same sedative or analgesic level [20].

The pharmacokinetic parameters of xylazine have been confirmed in various animal species [21]. Generally, the absorbance, metabolism and elimination of xylazine is quite rapid. It absorbs and spreads so quickly that the brain and kidney had the highest concentration of xylazine in organs a few minutes post-intravenous administration. The sedative effect can appear within 5 min and lasts up to 4 h [22,23]. The routine dose of xylazine in animals was 0.5–5.0 mg/kg. The highest concentration of xylazine in plasma occurs 0.2~0.3 h after intramuscular administration of 0.6–1.4 mg/kg in large animals such as sheep [24]. It was reported that less than 1% of the drug is excreted unchanged in the urine in the cow and about 8% in the rat [20].

## 3. Analytic Aspects

The past decade has provided an important impetus for LC-MS/MS technology and research. Due to the rather high selectivity and sensitivity of LC-MS/MS, better performance than other technology and reliable results it is a perfect method that can achieve the goal of various applications [25,26,27]. LC-MS/MS was successfully applied in pharmacology and toxicology cases and became an important instrument that cannot be ignored [25]. For one thing, pharmacology is the basis for drug monitoring and finding the correct treatment strategy for patients. Furthermore, LC-MS/MS are the most crucial instrument configurations used for drug identification and illicit drug screening in toxicology and forensic practice and research, providing enormous support to related legal practice. It is no wonder that LC-MS/MS play a critical role in the identification of xylazine.

In 2018, Krongvorakul et al. first established a LC-MS/MS method to detect xylazine in the serum and urine of the victims in drug-facilitated crimes and confirmed the concentration of serum (0.057 μg/mL) at 6 h and urine (0.294 μg/mL) at 8 h after she drank the drink [8]. In 2021, Xu et al. used LC-MS/MS to detect xylazine in the blood of 12 victims in a poisoning case; the minimum detection limit was 0.02 ng/mL and xylazine displayed a good linear relationship in the range of 0.1~200 ng/mL while the correlation coefficient R2 = 0.9978 and the blood concentration of xylazine in the victims ranged from 9.6 to 139.5 ng/mL [28]. In 2023, Yao et al. used LC-MS/MS to detect the potential drugs in 10 victims’ consumption of beef and venison; xylazine was detected in the blood and urine of the victims and rather high concentrations of xylazine were detected in the cooked beef and venison [29].

To better understand the current stage in the analysis method of xylazine, we identified several biological analysis methods of xylazine used in the past 10 years and summarize them in Table 1 [28,29,30,31]. LC-MS/MS, SPE-HPLC/MS/MS and UPLC-QTOF/MS were the validated methods in the detection of xylazine. Serum, blood and urine were common biological samples in the identification of xylazine as shown in Table 1. The LODa of the reported method was largely affected by the matrix, sample preparation and detection method. With the technology development of MS, the detection method revealed little difference in the identification of xylazine.

Blood and urine were common in the identification of xylazine in forensic practice. However, other organs such as the liver were rarely involved in such cases, and pre- and postmortem organ distributions of xylazine were seldom involved. Due to the occurrence of food poisoning, the left food was also an ideal sample in such cases to confirm the existence of xylazine.

Table 1 also indicates that the sample preparation of the validated methods was a traditional liquid–liquid extraction, which required large amounts of organic solvent and a complicated operation. The procedures of most methods were time-consuming, qualitative instead of quantitative, and needed a rather large sample size, and thus most of them were not suitable for the rapid detection of xylazine for clinical and forensic purposes. To date, few studies have centered on the tissue distribution of xylazine in the forensic perspective.

Although xylazine is emerging as an adulterant and is most commonly associated with other drugs including fentanyl, the metabolic pathways and major metabolites in humans who suffered from xylazine poisoning are absent. In 2015, Gao et al. established a UPLC-QTOF/MS method to detect the concentrations of xylazine and 2,6-xylidine (DMA) in the blood and urine [31]. In 2019, Cui et al. used the liquid chromatography-quadruped/orbitrap mass spectrometry method to explore the metabolites of xylazine in human urine and concluded that the hydroxylated products, oxidation products, S-oxidation products, etc., were the main metabolites of xylazine, which corresponds to the results of rat urine, rat liver microsomes, and horse urine [32]. The rather similar metabolites of xylazine in human urine in Chinese and Caucasian humans indicated the common metabolic pathways and major metabolites in different populations [33]. Previous studies also confirmed that xylazine undergoes phase I metabolic reactions such as hydroxylation, oxidation, N-dealkylation, and S-oxidation in the human body, and the hydroxylated metabolites then act on glucuronic acid and sulfuric acid to produce phase II metabolic reactions [32,33,34]. The main metabolite of xylazine, DMA, was a useful biomarker for post-poisoning surveillance.

## 4. Clinical and Forensic Aspect of Xylazine Poisoning

### 4.1. Characteristics of Xylazine Poisoning

To identify the characteristics of xylazine poisoning cases in clinical and forensic practice, we searched “Xylazine poisoning” in PubMed and CNKI, excluded the cases without humans involved, and identified 20 papers which included more than 160 cases; the related information is summarized in Table 2 and Table 3 [8,10,11,13,15,28,29,30,34,35,36,37,38,39,40,41,42,43,44,45]. As shown in Table 2, most of the involved cases were accidental; however, homicide and suicide were also seen in the xylazine poisoning cases. It was reported that it may occur in persons who accidentally ate meat that was injected with xylazine, and due to the rather small dose of xylazine, the victim may be rescued [28,29]. This was associated with mass poisoning incidents, and it may occur in homicide cases. In 2023, Xu et al. reported that a criminal purchased xylazine on the internet and poisoned the breakfast in a hotel; the scene investigation revealed that the criminal had been fired a few ago and he did this for revenge. Recently, injection was a common exposure of xylazine in the USA, and it was also combined with fentanyl or heroin in those illicit drug overdoses [3,4]. However, inhalation was a new approach in the exposure of xylazine and it may become popular as necrotizing skin ulceration has followed the injection. This related skin injury is different from the wound common in injection drug users and can occur at or away from the injection site regardless of the exposure route [3,4].

Coma, bradycardia and hypotension were the primary symptoms in xylazine poisoning cases and they may occur within 30 min and last for several days. Hypokalemia and small pupils were also reported. The time interval between xylazine exposure and the primary symptom in Table 2 was as soon as 15 min or as late as 2 h, and due to the unavailable information on the time interval between xylazine exposure and sample collected, the association link between clinical characteristics and blood or urine concentrations was hard to build. The signs and symptoms of xylazine poisoning are common to various disease states and/or toxicological exposures; therefore, xylazine cannot be detected rapidly in real time and can be a barrier to treatment, and should be added to the differential diagnosis if a toxicological cause is suspected.

Generally, central nervous system depression was common in such cases and respiratory depression was reported in people taking xylazine; the underlying mechanism may be attributed to the increase in the occurrence of respiratory depression induced by opioids. It is a common view that naloxone does work on respiratory depression caused by opioid whereas it does little to xylazine-induced respiratory depression [3,4]. However, xylazine poisoning may mimic an opioid overdose, and due to the prevalence of opioid overdoses, naloxone is still needed in such cases. Additional therapeutic support is required even if naloxone administration has been given. This additional therapeutic support includes keeping control of the airways, supplemental oxygen, and other treatment as needed. To date, there are still no approved drugs that can reverse the effect of xylazine in humans [3,4].

To date, the characteristics of xylazine poisoning have been so obscure that even experienced physicians may make incorrect diagnoses. However, some signs should arouse the attention of clinicians to the possibility of xylazine poisoning. When skin ulcers are seen without known reasons or naloxone has little effect on opioid overdose patients, or people who eat cooked beef and meat purchased from the same shop show coma and bradycardia, the clinicians should keep in mind the possibility of xylazine poisoning to make an early diagnosis. Once confirmed, oxygen-inhaling, naloxone therapy and symptomatic treatments were needed.

### 4.2. Characteristics in Xylazine-Related Death Cases

Generally, pure xylazine overdose death cases were uncommon in forensic practice. Although numerous studies indicated the increasing incidence of xylazine in forensic practice, most of them just focused on the detection method of xylazine and other characteristics of xylazine-related deaths and the pathological changes in such cases were rarely reported. The reasons may be the non-specific pathological changes in such cases, and xylazine contributes little to the cause of death in most of cases. Table 3 summarizes the forensic autopsy cases of xylazine overdose death. All the involved cases were homicide or suicide, and injection was the only exposure. The groin, right thigh and antecubital fossa were the injection sites; the bleeding injection site, bleeding point in the conjunctiva and congested edematous lung were non-specific and were also seen in other causes of death.

Although Sibbesen et al. retrospectively studied 3292 drug deaths from 2019 to 2021 in West Virginia and identified 117 cases involving xylazine, they found that xylazine-related death always involved other drugs such as opioids, stimulants, benzodiazepines, and antidepressants/antipsychotics. It also indicated that liver diseases were more common in xylazine-related deaths. Injection was still the leading exposure of xylazine in xylazine-related deaths. Other exposure routes include snorting, smoking; oral and inhaling were also reported.

Skin ulcers are a primary health problem in chronic xylazine users that arouse public attention. These skin ulcers can occur far from the injection site, which could distinguish them from other chronic drug users. The underlying mechanisms were attributed to the oxygenation response to xylazine intoxication of the skin, and the chronic use of xylazine may limit the mobility of the limbs and lead to amputation in severe cases [9,46,47,48,49,50]. Generally, the injection site in such cases should be examined carefully and distinguished from iatrogenic injection injuries. Therefore, it is critical to emphasize the scene investigation, case history and toxicological test results.

### 4.3. Mechanism of Xylazine-Related Death

In 2007, the abuse of xylazine in humans was first reported in Puerto Rico [51,52]. Since then, xylazine abuse has been recorded in other states (mainly in the Northeast), as well as an increasing number of drug-related deaths [53]. It was confirmed as a fentanyl adulterant as it prolonged the duration of fentanyl effects and increased the duration of brain hypoxia [51]. When used with opioids, xylazine has a synergistic effect and it could increase the level of brain hypoxia that may increase the risk of overdose or death [54]. Bradycardia, a depressed central nervous system, and hypotension were the most common side effects associated with human xylazine poisoning. To date, there is no clear definition of xylazine toxic in humans and the lethal concentrations of xylazine in humans have been absent. The role of xylazine in xylazine-related death is far from confirmed as xylazine and fentanyl were the common findings in such cases. The toxic mechanism of xylazine and the combination of xylazine and fentanyl still need further study to be confirmed.

### 4.4. Blood and Urine

#### Xylazine Concentrations

The exact contribution of xylazine to the process of death is still unknown, although its pharmacological effects were reported to enhance the respiratory and central nervous system depressive effects of other substances. The toxicity and lethal concentrations of xylazine are still unclear and there is considerable overlap in the blood concentration in patients who suffered xylazine poisoning and xylazine overdose death cases [1]. It is reported that the blood concentration of xylazine ranged from 5 to 49 ng/mL in xylazine overdose death cases, whereas blood or plasma concentrations ranging from 30 to 460 ng/mL were also detected in non-lethal xylazine overdose cases [1]. The concentration of xylazine summarized in Table 2 and Table 3 showed that the blood concentration of xylazine can reach 540 ng/mL in non-lethal xylazine overdose cases. Although there is not a significant difference in xylazine concentrations in the number of co-intoxicants, rather higher concentrations of xylazine were seen in decedents who took only one other drug compared to those who took more drugs. Indeed, xylazine plays an important role in the death mechanisms of other co-intoxicants.

Table 3 also shows that the blood concentration of xylazine in homicide cases was higher than in suicide cases, and was much higher in pure xylazine poisoning cases compared to combined with other toxics. To date, only a homicide case was reported and xylazine was the only reported toxic; whether the type of death and the cause of death had a role in the concentration of xylazine in different samples still needs further study. The blood concentration of xylazine was the lowest in most cases, while the urine concentration of xylazine was much higher. The urine concentration of xylazine can reach 533 ng/mL even in non-lethal xylazine overdose cases. The concentration of xylazine in the heart blood was lower than in the peripheral blood; considering the existence of postmortem redistribution in toxic overdose death cases, the heart blood may not be the most suitable sample in such cases. And due to the existence of putrefaction and autolysis, blood samples may be unable to be collected.

### 4.5. Alternative Samples

The organ and fluid distribution of xylazine in xylazine overdose death cases has rarely been reported in forensic practice. To date, only two research articles published the organ distribution of xylazine in xylazine overdose death cases with paradoxical results. In 1985, Poklis et al. reported that a 36-year-old male injected xylazine to commit suicide and the organ concentration of xylazine was as follows: lung (1100 ng/mL), liver (900 ng/mL), kidney (600 ng/mL), brain (400 ng/mL) [11]. In 2003, Moore et al. reported that a 42-year-old male injected xylazine to commit suicide and the concentration of xylazine in the kidney (780 ng/mL) was higher than in the liver (610 ng/mL) [45]. This may be attributed to the intake dose of xylazine as the intake dose of other drugs revealed the distribution characteristics in vivo are different in various lethal doses. Due to the existence of postmortem dispersion and other factors, the postmortem redistribution of xylazine was an inevitable phenomenon.

The affected factors included diffusion distance, postmortem gavage time and dose. It is prone to disperse into tissues and organs close to the gastrointestinal tract, whereas it did have some impact on the heart blood, bile, and peripheral blood, and never affected the brain and muscles. Therefore, to identify the concentration of xylazine in forensic practice, not only the routine examination materials in forensic toxicological practice such as the heart blood, liver and stomach content should be collected, but also the less affected tissues and organs such as the brain and muscle should be taken for examination.

When it comes to the xylazine overdose death cases whose exposure route was injection, the injection site was the ideal sample for the detection of xylazine. Therefore, it should be carefully examined, taken fixed in formalin for histopathological examination and taken stored at −80 °C for subsequent analysis. Generally, the routine sample for the detection of xylazine was not identified in forensic practice, and it still needs further study to confirm the ideal sample in such cases.

### 4.6. Other Drugs or Toxins in Such Cases

It has been reported that in almost all xylazine deaths, 98.3%, the patient also took fentanyl, and most of the decedents were young white men [9]. Previous studies have documented this high fentanyl prevalence, consistent with the identification of xylazine as a fentanyl adulterant [46,47,48,49,50]. Xylazine is increasingly involved in drug overdose decedents that ingest more toxicants, and xylazine abuse is an emerging public health problem [46,47,48,49,50].

It has been reported that drug or alcohol abuse history increases the incidence of xylazine-related death [9]. Xylazine-related deaths are more common in individuals who take more than two drugs, including xylazine. In 2022, Sibbesen et al. retrospectively reviewed 3292 drug deaths in West Virginia from 2019 to mid-2021, identified the characteristics of xylazine-related death, found that approximately 90% of the xylazine-related decedents took more than three drugs, and that 40% of the xylazine-related decedents had taken more than five drugs [9]. Opioids, stimulants, benzodiazepines, and antidepressants/antipsychotics are commonly associated with xylazine-related deaths. Fentanyl analogues, heroin and other opioids are found in about a quarter of xylazine-related deaths and nearly one in five of non-xylazine deaths.

Few studies focus on the interaction between xylazine and fentanyl. In 2023, Smith et al. used C57BL/6 mice which were 42 days of age to quantify the lethal effects of fentanyl and xylazine, found that 56 mg/kg fentanyl administration could shift the LD50 of xylazine from 157.2 mg/kg to 32.0 mg/kg and 100 mg/kg xylazine administration could shift the LD50 of fentanyl from 131.3 mg/kg to 1.27 mg/kg, suggesting the synergistic manner in their interaction and that the combination of them can lead to more rapid death than alone [54]. Choi et al. used adult male Long–Evans rats that weighed 450 ± 50 g to evaluate the neuro effects of xylazine alone or combined with fentanyl and heroin and found that xylazine alone not only can decrease temperature and locomotor activity, but also prolong the lower oxygen levels in the brain [55].

The existence of other drugs complicates the role of xylazine in xylazine-related death in forensic practice. The concentration of other drugs may be far from the lethal concentration, but xylazine may enhance the toxic effect of them, and result in unexpected death. However, few studies focused on the interactions between them, and other drugs may affect the metabolism of xylazine, which needs further study.

## 5. Future Challenges

Due to the characteristics of xylazine and the influence of the internet, xylazine abuse is increasing in clinical and forensic practice, and the identification of such cases is still a challenge. Therefore, we summarized the updated information about xylazine and xylazine poisoning cases and thus provide a reference and give advice on future research.

Firstly, we summarized the characteristics of xylazine poisoning cases in clinical and forensic practice and thus provided reference in clinical and forensic practice. Secondly, the detecting method of xylazine and samples (tissues and liquid) were also listed to update toxic information about xylazine and thus throw light on further study. Thirdly, other drugs or toxicants may also be detected in xylazine poisoning cases, which may complicate such cases in clinical and forensic practice and need further study to make clear their interactions.

Lastly, the frequency of illicit drug users who use xylazine has exceeded the scope of accidental drug outbreaks. It is speculated that it may be attributed to the spread of internet usage in illicit drug users, and the ease of obtaining the drug from the internet. It is a common view that xylazine can be used as a veterinary anesthetic. The over-the-counter availability and low price of xylazine provides the victims with a convenient way to obtain it. Therefore, public health agencies should consider focus on the education of xylazine poisoning and monitor the real-time information on xylazine poisoning [56].

## 6. Conclusions

Above all, although we update the latest information about xylazine poisoning, the exact lethal concentration of xylazine is still vague; many factors, such as the age of the victim, pre-existing morbidity and co-exposure, complicate the critical role of xylazine in xylazine poisoning cases. Therefore, the concentration of xylazine and its role in the symptoms of xylazine poisoning cases still need further study to confirm. Furthermore, the combined drugs in xylazine-related poisoning cases complicate the role of xylazine in clinical and forensic practice and the interaction between xylazine and other drugs was seldom involved in the present studies. And lastly, the organ distribution and toxic mechanism of xylazine poisoning still needs further study to be confirmed and we hope more clinical and forensic pathologists and toxicologists can join us and make it clear.

## Figures and Tables

**Table 1:** Some analytic methods of xylazine and metabolites (DMA) used in biological samples.

	Analysis	Matrix (mL)	Sample Preparation	Sample	LODa ng/mL	Linear Range ng/mL	Author, Publication Year
Xylazine	LC-MS/MS	1.0 2.0 g	Liquid—liquid extraction	Blood, urine, beef, venison	Blood 2.5, urine, 2.5, Meat 2.5 µg/kg	1.0~300.0	Yao et al. 2023, [29]
	LC-MS/MS	1.0	Liquid—liquid extraction	Blood	0.02	0.1~200	Xu et al. 2023, [28]
	LC-MS/MS	0.5	Liquid—liquid extraction	Serum, Urine	Serum, 1, Urine 1	Blood 10~750, Urine 10~750	Krongvorakul et al. 2018, [8]
	SPE-HPLC/MS/MS	1.0	SPE	Blood, Urine	Blood 0.4, Urine 0.3	Blood 2~2000, Urine 2~2000	Liu et al. 2017, [30]
	UPLC-QTOF/MS	1.0	Liquid—liquid extraction	Blood, Urine	Blood 0.4, Urine 0.3	Blood 2~2000, Urine 2~2000	Gao et al. 2015, [31]
DMA	SPE-HPLC/MS/MS	1.0	SPE	Blood, Urine	Blood 0.4, Urine 0.3	Blood 2~2000, Urine 2~2000	Liu et al. 2017, [30]
	UPLC-QTOF/MS	1.0	Liquid—liquid extraction	Blood, Urine	Blood 0.5, Urine 0.3	Blood 10~4000, Urine 10~4000	Gao et al. 2015, [31]

Matrix (mL): minimal sample size.

**Table 2:** Xylazine poisoning cases in clinic and forensic practice without autopsy details.

Authors and Publication Year	Age/Sex	Cause	Exposure Route	Primary Symptoms	Time Interval between Oral and Symptom	Drug Concentration
Yao et al. 2023, [29]	Unknown, 5 victims	Accident	Oral	Drowsiness, hypokalemia	15–40 min Mean 25 min	Blood 4.8–11.3 ng/mL, Urine 6.7–218.1 ng/mL, Cooked beef 440.0 µg/kg
	Unknown, 5 victims	Accident	Oral	Dry mouth, dizziness, drowsiness	30–60 min Mean 45 min	Blood 8.6–46.8 ng/mL, Venison 193.0 µg/kg Cooked venison 5.0 mg/kg
Xu et al. 2023, [28]	Unknown, 12 victims	Homicide	Oral	Dizzy	Unknown	9.6~139.5
Gill EL et al. 2023, [34]	8/M	Unknown	Unknown	loss of consciousness, hypopneic with pinpoint pupils, headache	Unknown	Xylazine and fentanyl positive
Krongvorakul et al. 2018, [8]	73/F	homicide	Oral	Coma, bradycardia	Unknown	Gastric content, positive. Serum, negative
	71/F	homicide	Oral	Coma, bradycardia	Unknown	Urine, 0.294 μg/mL Serum, 0.0057 μg/mL
	76/M	homicide	Oral	Coma, bradycardia	Unknown	Urine, 0.533 μg/mL
Liu et al. 2017, [30]	unknown	Accident	Oral	Unknown	Unknown	Peripheral blood xylazine and 2,6-Dimethylaniline positive
Forrester MB. 2016, [35]	76 victims, 41M, 33F, mean age 37	49 unintentional, 24 were intentional, 1 misuse; 1 adverse reaction, 1 unknown	39 injection, 21 ingestion, 12 dermal route, 11 ocular route, 2 inhalation, and 2 unknown, 9 multiple.	Drowsiness/lethargy [36], Bradycardia [15], Hypotension [8], Hypertension [7] Puncture/wound [6], Slurred speech [6], Coma [5], Ocular irritation/pain [5], Respiratory depression [4]	Unknown	Positive
Wang et al. 2014, [36]	Unknown, 1 male, 4 females	Suicide	Oral	Unknown	Unknown	200–1000 mg, one death
Hou et al. 2013, [37]	Unknown, more than 50 victims	Accident	Oral	Unknown	Unknown	Peripheral blood, cooked meat xylazine positive
Meyer et al. 2013 [38]	14/M	Accident	Injection	Coma, bradycardia	Unknown	Plasma 0.3 mg/L of xylazine and 0.1 mg/L of ketamine
Zhang et al. 2010, [39]	31/M	Accident	Inhaled	Dyspnea	Unknown	Unknown
Liu et al. 2007, [40]	19/M	Accident	Oral	Coma, 35.5 °C, bradycardia	Unknown	Urine 582 mg/L of ketamine, 448 mg/L of norketamine, 745 mg/L of phenobarbital, and 762 mg/L of xylazine
Velez et al. 2006, [13]	38/M	Accident	Unintentional irrigation of both eyes	Bradycardia, hypotension to 90/60 mm Hg, consciousness impairment,	2 h	Unknown
Xia et al. 2006, [41]	7 victims, 1 male, 6 females, 28–48 years old	homicide	Oral	Dizziness, drowsiness, palpitations, impaired consciousness	0.5–2 h	Heart blood 12.86 μg/mL
Elejalde et al.2003, [42]	18/M	Suicide	inhaled	Blood pressure 90/60 mmHg, heart rate 45 bpm, disorientation, dysarthria, dysmetria, ataxia, sinus bradycardia	Unknown	urine for common toxins was negative
Capraro et al. 2001, [15]	16/M	Suicide	inhaled	Coma, bradycardia	Unknown	Blood 0.54 μg/mL perhaps 3 mg of dose
Samanta et al. 1990, [43]	19/M	Accident	Injection	80/60 mm Hg, small pupils	30 min	Unknown

**Table 3:** Xylazine poisoning cases in forensic practice with forensic autopsy.

Authors and Publication Year	Age/Sex	Cause	Exposure Route	Primary Symptoms	Time Interval between Oral and Symptom	Time Interval between Oral and Death	Autopsy and Pathology Findings	Postmortem Sampled Time	Postmortem Drug Concentration ng/mL
Rock et al. 2023, [10]	43/F	Suicide	Injection	Unknown	Unknown	Unknown	Recent puncture wounds to the groin	Unknown	Blood 38 Urine 135
Zhang et al. 2021, [44]	49/F	Homicide	Injection	Dizzy	30 min	Less than 10.5 h	Bleeding point is seen in the conjunctiva, bleeding was observed on the injection site of right thigh	Unknown	Heart blood 2.4 Injection site positive
Moore et al. 2003, [45]	42/M	Suicide	injection	Unknown	Unknown	Unknown	Unknown	Unknown	Heart blood 2300 ng/mL, peripheral blood 2900, Bile 6300, Kidney 7.8 mg/kg, Liver 6.1 mg/kg, Urine 10
Poklis et al. 1985, [12]	36/M	Suicide	Injection	Unknown	Unknown	Within 4 h	Injection sites on antecubital fossa, congested edematous lung	Unknown	Blood 200, Brain 0.4 mg/kg, Kidney 0.6 mg/kg, Liver 0.9 mg/kg, Lung 1.1 mg/kg, Adipose 0.05 mg/kg, Urine 7000

## Data Availability

Not applicable.
